# Investigating mammary glands of lactating goats for the presence of tertiary lymphoid organs

**DOI:** 10.3389/fimmu.2022.941333

**Published:** 2022-08-10

**Authors:** Yusaku Tsugami, Sayaka Nakayama, Naoki Suzuki, Takahiro Nii, Naoki Isobe

**Affiliations:** Graduate School of Integrated Sciences for Life, Hiroshima University, Hiroshima, Japan

**Keywords:** immunoglobulin, mammary glands, mastitis, tertiary lymphoid organs, tight junctions

## Abstract

Ectopic tertiary lymphoid organs (TLOs) have been identified in many organs, such as the lungs, nasal cavities, and kidneys of both mice and humans. Although lymphocyte aggregates have been observed in the mammary glands of ruminants, the details remain unclear. In this study, we investigated the mammary glands of lactating goats for the presence of TLOs. The localization of CD20 (B cells), CD3 (T cells), MECA79 (high endothelial venules), CD40 (follicular dendritic cells), BCL6 (germinal center), and IgA was examined by immunohistochemistry. The concentrations of IgG, IgA, lactoferrin, β-defensin-1, cathelicidin-2, cathelicidin-7, S100A7, and S100A8 in milk were measured by ELISA. The localization and amount of tight junction (TJ) proteins (claudin-3 and claudin-4) were examined using immunofluorescence and western blotting. We found that 19 out of 30 udders contained lymphocyte aggregates, which showed positive reactions against CD20, CD3, CD40, and MECA79. In addition, large-sized aggregations showed separate localization of B cells and T cells and a positive reaction against BCL6, although BCL6 was sparsely localized in the aggregations. These results indicate that mammary glands of lactating goats contain TLOs. The IgG and IgA concentrations in the milk of TLO-positive goats and the number of IgA-positive cells were higher than those in negative goats. Furthermore, claudin-4 was localized in the TJ region and the amount was higher in TLO-positive mammary glands than that in the negative group, indicating the presence of leakages at TJs. In conclusion, a majority of lactating goat udders have TLOs, which contribute to local immunity by producing immunoglobulins.

## Introduction

Mastitis decreases milk production and causes large economic losses in the dairy industry (1). To prevent mastitis, vaccination against mastitis-causing pathogens such as *Escherichia coli* and *Staphylococcus aureus* is considered effective (2, 3). Killed bacteria are commonly used as inactivated vaccines in dairy cows. Intramuscular vaccines induce an increase in antigen-specific IgG in the blood, and mucosal vaccines induce an increase in antigen-specific IgA in mucosal tissue. Secondary lymphoid organs, such as the spleen, lymph nodes, and Peyer’s patches, regulate the production and class switching of immunoglobulins. In secondary lymphoid organs, B cells proliferate at the germinal center and mature through the presentation of antigens by dendritic cells or T cells.

Recently, ectopic lymphoid tissues, besides secondary lymphoid organs, have been reported in many organs, such as the lungs (4), nasal cavities (5), and kidneys (6) of mice and humans. These ectopic lymphoid tissues are also called tertiary lymphoid organs (TLOs) and provide local immunity by producing immunoglobulins. Various authors have defined the development of TLOs. Jamaly et al. classified the TLOs in kidneys into three stages: immature, arrangement of T cells and fibroblasts; premature, presence of follicular dendritic cells, mature dendritic cells, and small B cell follicle; and mature, high endothelial venules (HEVs) and formation of the germinal center (6). Schumacher and Thommen classified TLOs in cancer into three stages: stage 1, arrangement of T cells and B cells with HEVs; stage 2, presence of follicular dendritic cells and small B cell follicle; and stage 3, formation of the germinal center (7). Dieudé et al. classified the TLOs into three stages: stage 1, arrangement of T cells and B cells with follicular dendritic cells; stage 2, B cell clusters surrounded by T cells; and stage 3, presence of HEVs and formation of the germinal center (8). Therefore, in this study, we investigated the TLOs in mammary glands classified into three stages: stage 1, arrangement of T cells and B cells with follicular dendritic cells; stage 2, separate localization of B cells and T cells; and stage 3, formation of the germinal center.

Immunoglobulins in milk, such as IgG and IgA, inhibit the growth of pathogens and the invasion of antigens into the host body through bacterial agglutination and toxin neutralization (9). Similar to immunoglobulins, various antimicrobial components also work to defend the host from pathogens. Lactoferrin has a bacteriostatic effect decreasing the iron availability for microorganisms (10). Defensins and cathelicidins disrupt the target microbial membranes and contribute to the killing of pathogens (11, 12). The S100 protein family contains EF-hand helix-loop-helix domains that help them to function as calcium-binding proteins (13). However, the interaction with TLOs and other antimicrobial components remains unclear.

In the intestine, the constituent proteins of tight junctions (TJs) differ between the villous epithelium and the follicle-associated epithelium of Peyer’s patches (14). TJs seal the paracellular pathway between the epithelial cells. The claudin family is a constituent protein of TJs, which regulates the permeability of ions, water, and small molecules via paracellular pathways (15). The permeability of TJs depends on the claudin subtype at the TJ strands. In the intestinal villous epithelium of rats, claudin-3 and claudin-8 are localized at the most-apical region of lateral membranes, whereas claudin-4 is localized at the follicle-associated epithelium of Peyer’s patches (16). In lactating mammary glands of both rodents and ruminants, less-permeable TJs composed of claudin-3 prevent the mixing of milk components with blood components and the invasion of pathogens into the host body (17). In contrast, the TJ barrier in mammary glands with inflammation is weakened by an increase in claudin-4 expression in mice and cows (18, 19).

The aggregation of lymphocytes, such as TLOs, has been observed in the mammary glands of ruminants (20–22). However, the feature or stage of these TLOs, the production of immunoglobulins and their interaction with other antimicrobial components, and the properties of TJs in mammary epithelial cells near the TLOs remain unclear. If TLOs exist in mammary glands, they may be able to induce specific immunoglobulins against mastitis-causing pathogens locally and thereby effectively prevent mastitis. Thus, in this study, we investigated the presence or absence of TLOs in the mammary glands of lactating goats.

## Materials and methods

### Experimental animals

In this study, we used seven Tokara (26.4–37.4 kg, 2–7 years old, 1–6 parity) and ten Shiba goats (14.8–39.4 kg, 2–6 years old, 1–7 parity) at the lactation stage for milk and tissue collection. The goats were individually housed under a 14:10 h light:dark cycle, were fed 0.6 kg of hay cubes and 0.2 kg of barley per day, and had free access to water and trace-mineral salt blocks. Feed was offered twice daily at 08:00 and 15:00 h. The diet (energy, protein, and minerals) for the goats was calculated according to the Japanese feeding standard for sheep (Ministry of Agriculture, Forestry, and Fisheries in Japan, 1996) (23). Milking was performed by hand once daily at 08:00 h. All goats consumed their feed completely, did not show any symptoms of fever and diarrhea, and did not have mastitis. In addition, most goats were previously used for research on *E. coli* (heat-killed *E. coli* O111:B4, In vivoGen, 10^8^ cells/udder) or lipopolysaccharide (LPS, O111, FUJIFILM Wako Pure Chemical Corporation, 0.1–5 μg/udder) infusion at this or previous lactating term, as shown in **Table 1**. The goats (infused at this lactating term) were infused toxins more than 3 weeks before tissue collection. We confirmed the increase in somatic cell count (SCC) or cytokine concentrations in milk after toxin infusion, although the cell counts or cytokine concentrations returned to normal within a week. All experiments were approved by the Animal Research Committee of Hiroshima University (No. C14-5) and were conducted according to the Guidelines for Animal Experiments at Hiroshima University.

**Table 1:** Description of experimental goats used in the study.

Breed	ID/side	birth	parturition	sampling	parity	- or +	LPS or *E. coli* infusion	
							This	Previous
**Shiba**	1/L	2017/2/27	2020/11/16	2021/2/3	4	–	*E. coli*	–
	1/R					+++	*E. coli*	–
	2/L	2017/12/19	2020/11/16	2021/2/3	3	–	*E. coli*	1 μg LPS
	2/R					+++	*E. coli*	1 μg LPS
	3/L	2017/12/19	2020/4/16	2021/7/12	2	++	–	1 μg LPS
	3/R					++	–	1 μg LPS
	4/L	2018/3/28	2020/11/27	2021/3/25	2	+++	–	–
	5/L	2018/8/24	2020/12/23	2021/4/29	2	++	–	1 μg LPS
	5/R					–	–	1 μg LPS
	6/L	2019/3/14	2020/11/1	2021/5/25	1	+++	0.1 μg LPS	–
	6/R					+++	0.1 μg LPS	–
	7/R	2019/6/3	2020/11/27	2021/4/12	1	–	*E. coli*	–
	8/L	2019/11/21	2020/11/30	2021/4/29	1	–	*E. coli*	–
	8/R					–	*E. coli*	–
	9/L	2020/5/19	2021/5/5	2021/9/7	1	–	*E. coli*	–
	9/R					–	*E. coli*	–
	10/L	2015/3/4	2020/11/9	2021/1/28	7	++	*E. coli*	–
	10/R					++	*E. coli*	–
**Tokara**	11/L	2014/3/1	2021/3/11	2021/5/13	6	++	0.1 μg LPS	–
	12/L	2017/3/2	2021/4/15	2021/6/15	1	+	5 μg LPS	–
	12/R					–	5 μg LPS	–
	13/L	2017/3/2	2021/4/22	2021/5/21	1	+	–	–
	13/R					–	–	–
	14/L	2017/7/21	2021/4/27	2021/12/21	1	+	*E. coli*	–
	14/R					+++	*E. coli*	–
	15/L	2017/12/12	2021/6/1	2021/9/7	1	++	*E. coli*	–
	15/R					++	*E. coli*	–
	16/L	2018/4/25	2021/5/7	2021/8/6	2	–	0.1 μg LPS	–
	16/R					+	0.1 μg LPS	–
	17/L	2019/3/11	2021/3/29	2021/5/25	1	++	0.1 μg LPS	–

−, lymphocyte aggregation-negative group. +, lymphocyte aggregation-positive group and classification by the number of aggregations per udder (+; number of aggregations = 1, ++; 2 ≤ number of aggregations < 10, +++; number of aggregations ≥ 10). *E. coli* (O111:B4) at 10^8^ cells or lipopolysaccharide (LPS, O111) at each concentration were infused into mammary glands in this or previous lactating term.

### Tissue collection

The deep (mammary alveoli) and shallow (mammary cisterns) areas of the mammary gland tissues were collected from at least two places per udder, as previously reported (24). Deep sedation and anesthesia were achieved through slow intravenous injection of xylazine (Bayer HealthCare Pharmaceuticals Inc.) and pentobarbital (Somnopentyl; Kyoritsu Seiyaku), respectively, after which the goats were euthanized by exsanguination.

### Analysis of milk samples

Milk samples collected immediately before tissue collection were centrifuged at 5,000 × g for 10 min at 4°C. Milk fat and skim milk were separated from somatic cell pellets. The cell pellet was resuspended in PBS to determine the SCC, which was measured using a Countess II FL Automated Cell Counter (Thermo Fisher Scientific), as reported previously (24). Skim milk was stored at −30°C for conducting ELISA. The Na^+^ concentration in milk was measured using a LAQUAtwin Na-11 pocket meter (Horiba Ltd.).

### Immunohistochemistry and immunofluorescence

The collected mammary gland tissues were fixed, dehydrated, and embedded in paraffin. Sections (3-μm thick) were air-dried on MAS-coated slides. After deparaffinization, the sections were immersed in 0.3% hydrogen peroxide in methanol to inactivate endogenous peroxidases for immunohistochemistry. After being washed with PBS, antigen retrieval was then performed by autoclaving the sections in a citric acid buffer (pH 6.0) for 20 min at 121°C. Sections were washed with PBS for 10 min and incubated in PBS-T (PBS containing 0.05% Tween-20) containing 5% bovine serum albumin (BD Biosciences) for 1.5 h at room temperature. The sections were then incubated overnight at 4°C with either rabbit polyclonal antibodies against goat-IgA (#A50-106A, Bethyl Laboratories), claudin-3 (#34-1700, Thermo Fisher Scientific), claudin-4 (#PA5-32354, Thermo Fisher Scientific), or mouse monoclonal antibodies against BCL6 (#sc-7388, Santa Cruz Biotechnology), CD3 (#NBP2-53386, Novus Biologicals), CD20 (#NBP2-45454, Novus Biologicals), CD40 (#sc-13128, Santa Cruz Biotechnology), occludin (#sc-133256, Santa Cruz Biotechnology), or a rat monoclonal antibody against MECA79 (#sc-19602, Santa Cruz Biotechnology) diluted in PBS-T containing 2.5% bovine serum albumin. The lymph nodes were used as positive control for each marker of lymphoid organs (**Supplementary Figure 1**).

To identify the immunoreaction products from the immunohistochemical analysis, the sections were incubated with peroxidase-labeled goat anti-rabbit IgG and anti-mouse IgG antibody (Histofine MAX-PO, Nichirei Bioscience) or biotin-labeled rabbit anti-rat IgG antibody (Vector Laboratories) for 1 h at room temperature, followed by conjugation with peroxidase-labeled streptavidin (Hycult Biotech) for 30 min at room temperature. The immunosignals of the sections were visualized by incubation with a diaminobenzidine reaction mixture. The sections were counterstained with hematoxylin, dehydrated, and covered. For immunofluorescence, the sections were incubated with secondary antibodies (Alexa Fluor 488-conjugated goat anti-rabbit, #A32731; Alexa Fluor 555-conjugated goat anti-mouse, #A32727; Thermo Fisher Scientific) diluted with PBS-T containing 2.5% bovine serum albumin for 1 h at room temperature. Immunohistochemical images were obtained using the Eclipse E400 microscope and Digital Sight DS-Fi1 camera (Nikon), and immunofluorescence images were obtained using a fluorescence microscope (BZ-9000) and processed using analysis software (Keyence). A minimum of four different images (0.47 × 0.63 mm) per section were photographed and used to measure the frequency of IgA-positive cells, as reported previously (25).

### ELISA

Competitive ELISA was performed to measure the levels of lactoferrin, β-defensin-1, cathelicidin-2, cathelicidin-7, S100A7, and S100A8, as reported previously (26–28). Sandwich ELISA was performed to measure IgG and IgA levels by using rabbit polyclonal antibodies against goat-IgG (#A50-104A, Bethyl Laboratories), goat-IgG antibody-horseradish peroxidase (#A50-104P, Bethyl Laboratories), goat-IgA (#A50-106A, Bethyl Laboratories), and goat-IgA antibody-horseradish peroxidase (#A50-106P, Bethyl Laboratories). To measure *E. coli*-specific IgG and IgA, heat-killed *E. coli* O111:B4 (In vivoGen) was coated on a 96-well plate. After reaction with the milk samples, the titer was detected using rabbit polyclonal antibodies against goat-IgG or goat-IgA antibody-horseradish peroxidase. Sample dilutions yielding optical density readings in the linear portion of the appropriate standard curve were used to quantify the levels of each protein, except for *E. coli*-specific IgG and IgA. Standard and sample dilutions were added to each ELISA plate in duplicate or triplicate wells, and the optical density was measured using a microplate reader (Multiskan FC Microplate type 357; Thermo Fisher Scientific). The ELISA used was specific to goats.

### Western blotting

Western blotting was performed, as previously reported (29), with some modifications. Mammary gland tissues were lysed in a radioimmunoprecipitation assay buffer (25 mM Tris-HCl [pH 7.6], 150 mM NaCl, 1% NP-40, 1% sodium deoxycholate, 0.1% sodium dodecyl sulfate, and protease inhibitors) after being washed with PBS to remove milk. The lysates were lysed in a Laemmli sodium dodecyl sulfate-solubilizing buffer and then heated for 15 min at 70°C. The samples were separated on sodium dodecyl sulfate polyacrylamide gels and transferred to polyvinylidene difluoride membranes (Bio-Rad Laboratories). Immunosignals were detected using claudin-3, claudin-4, α-tubulin (#GTX628802, GeneTex, Los Angeles, CA, USA), secondary horseradish peroxidase-conjugated anti-rabbit antibody (Abcam), anti-mouse antibody (Sigma-Aldrich), and Immobilon Forte Western HRP Substrate (Millipore). Images of the bands were obtained using an Ez-Capture II (Atto). For quantification, the bands were analyzed using a CS Analyzer 3.0 (Atto).

### Statistical analysis

Data were expressed as the mean ± SD. Statistical analyses were performed using the SAS software (version 9.4, SAS Institute Inc.). The significance of differences was assessed using one-way analysis of variance with a post-hoc Student’s t-test. Differences were considered statistically significant at p < 0.05. The udders were randomly collected. The lymphocyte aggregation-positive and negative groups were divided after the observation of HE-stained sections of mammary gland tissues.

## Results

### Localization of each marker for TLOs in lymphocyte aggregations

**Figure 1:**
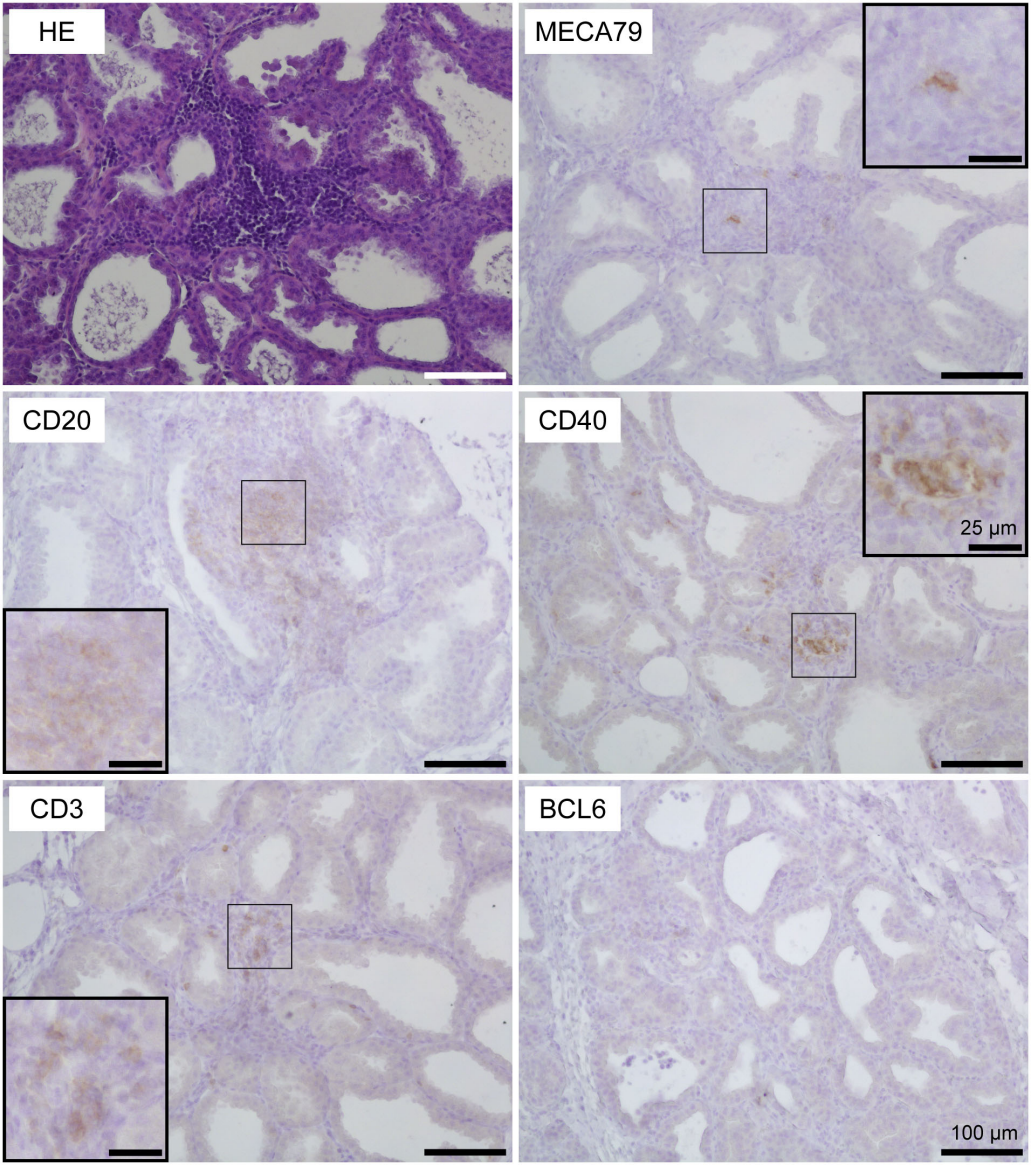
Representative images of hematoxylin and eosin staining (HE) and immunohistochemistry against CD20 (B cells), CD3 (T cells), MECA79 (high endothelial venules; HEVs), CD40 (follicular dendritic cells), and BCL6 (germinal center) in a small-sized aggregation of lymphocytes in goat mammary gland tissues. Scale bar, 100 μm or 25 μm (clippings).

**Figure 2:**
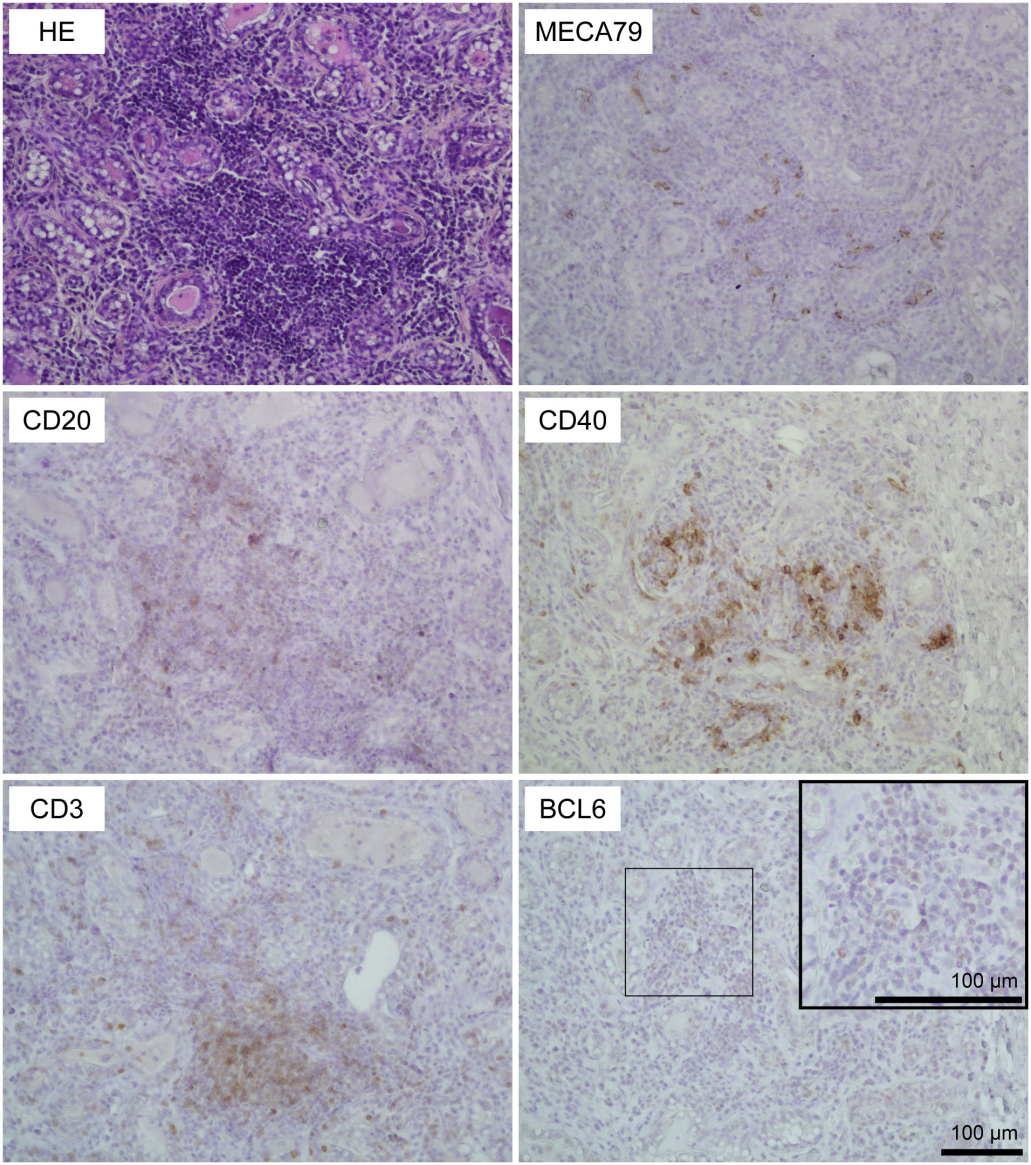
Representative images of hematoxylin and eosin staining (HE) staining and immunohistochemistry against CD20 (B cells), CD3 (T cells), MECA79 (high endothelial venules; HEVs), CD40 (follicular dendritic cells), and BCL6 (germinal center) in a large-sized aggregation of lymphocytes in goat mammary glands. Images of the same position are shown. Scale bar, 100 μm.

**Figure 3:**
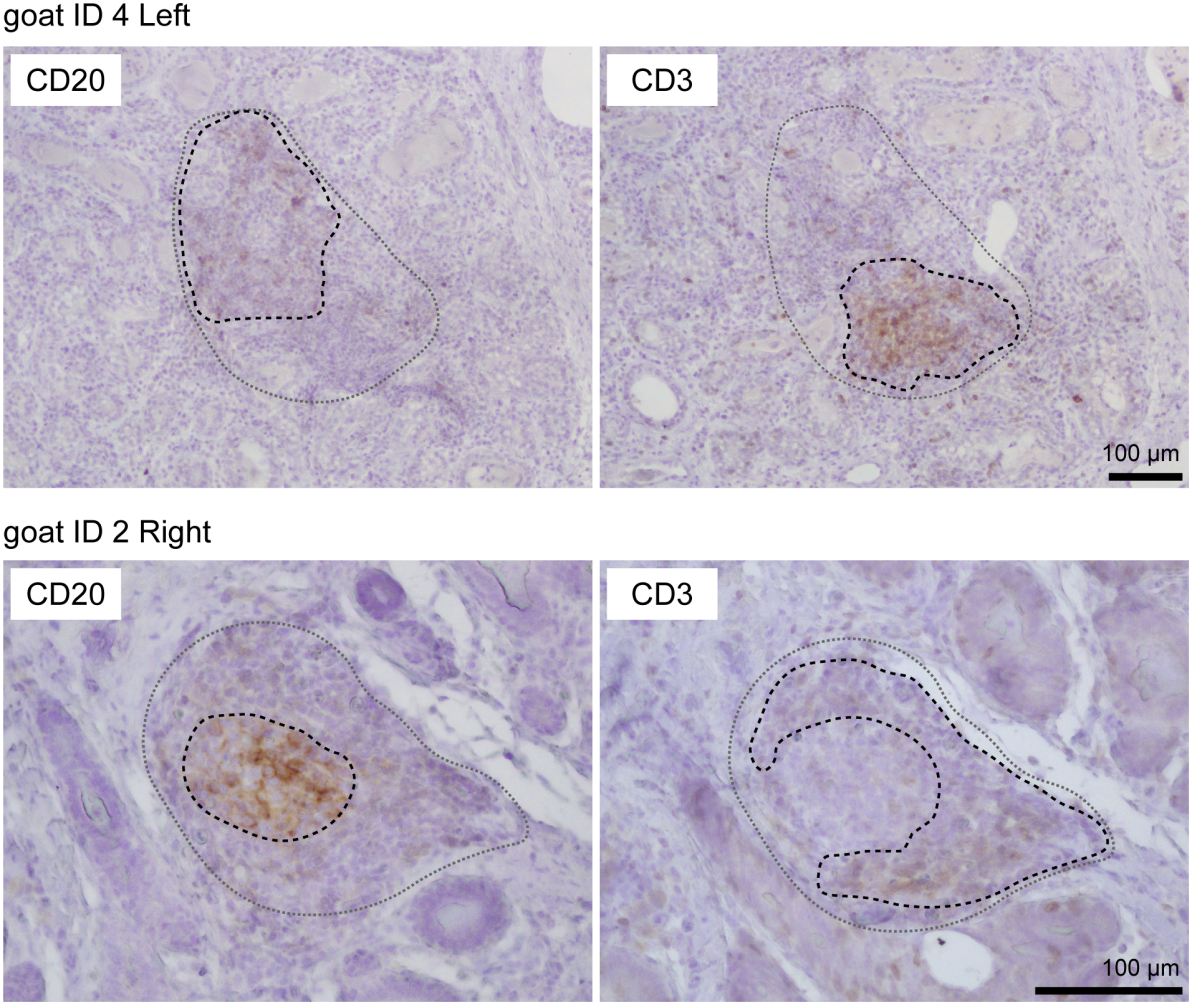
Representative images of immunohistochemistry against CD20 (B cells) and CD3 (T cells) in large aggregations of lymphocytes in goat mammary glands. Images of the same position are shown. Scale bar, 100 μm.

Using HE-stained sections of mammary gland tissues, we observed the aggregations of lymphocytes in the stromal region near both mammary alveoli and cisterns in 19 out of 30 udders. **Table 1** shows information regarding the experimental animals. The aggregations of lymphocytes were observed regardless of udder side. We carried out immunohistochemical analysis to investigate the localization of B cells (CD20), T cells (CD3), HEVs (MECA79), follicular dendritic cells (CD40), and the presence of a germinal center (BCL6) in the mammary glands of the goats (**Figures 1**, **2**). In small-sized aggregations of lymphocytes, a positive reaction against CD20 was observed over a wide range of the aggregation (**Figure 1**). CD3 and CD40 were collectively localized in the aggregates, in addition to a single localization near the mammary alveoli. We observed sparse positive reactions against MECA79 in the aggregation. A clear positive reaction against BCL6 was not observed in small-sized aggregations of lymphocytes. Our results showed positive reactions against CD20, CD3, MECA79, and CD40 in large-sized aggregations of lymphocytes (**Figure 2**), similar to those detected in small-sized aggregations. In addition, we also observed weak positive reactions against BCL6 during the aggregation. Furthermore, it appeared that there existed separate CD20 and CD3 zones in some lymphocyte aggregates (**Figure 3**).

### Influence of lymphocyte aggregation on IgG and IgA production in mammary glands

**Figure 4:**
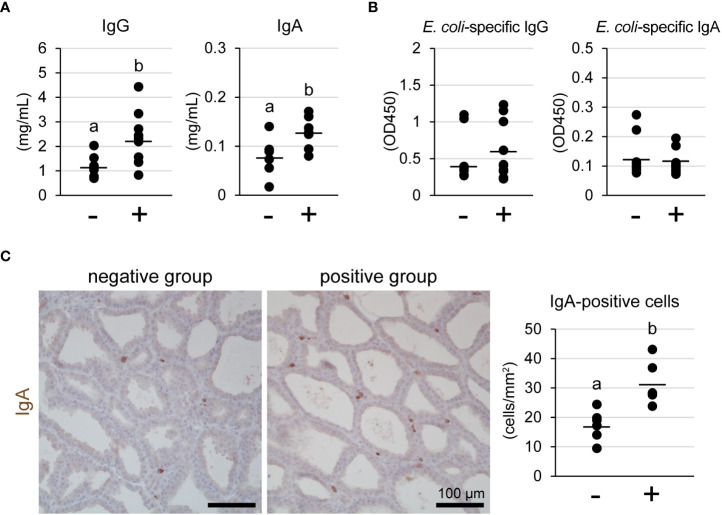
Concentrations of IgG and IgA **(A)** and titer against *Escherichia coli*-specific IgG and IgA **(B)** in milk of tertiary lymphoid organ-positive and -negative goats. **(C)** Representative images of immunohistochemistry against IgA and number of IgA-positive cells. (−) lymphocyte aggregation-negative group; (+) lymphocyte aggregation-positive group. Scale bar, 100 μm. Different letters indicate a significant difference between groups (p < 0.05).

We measured the concentrations of IgG and IgA in milk using ELISA (**Figure 4A**). The concentrations of both IgG and IgA in the lymphocyte aggregation-positive group (n = 12) were significantly higher than those in the negative group (n = 8) [IgG: 2.28 ± 0.78 mg/mL vs. 1.15 ± 0.48 mg/mL, p = 0.028; IgA: 128.6 ± 28.6 μg/mL vs. 78.3 ± 41.1 μg/mL, p = 0.015, respectively]. In contrast, the titers against *E. coli*-specific IgG and IgA did not show a statistical difference between the groups (**Figure 4B**). We determined the number of IgA-producing cells in the mammary glands using immunohistochemistry (**Figure 4C**). IgA-positive cells were observed in the stromal regions of the mammary glands, regardless of the presence of lymphocyte aggregation. However, the number of IgA-positive cells was significantly higher in the lymphocyte aggregation-positive group (n = 6) than that in the negative group (n = 6) [31.3 ± 7.2 cells/mm2 vs. 17.4 ± 5.1 cells/mm2, p = 0.003, respectively].

### Influence of lymphocyte aggregation on TJ proteins in mammary glands

**Figure 5:**
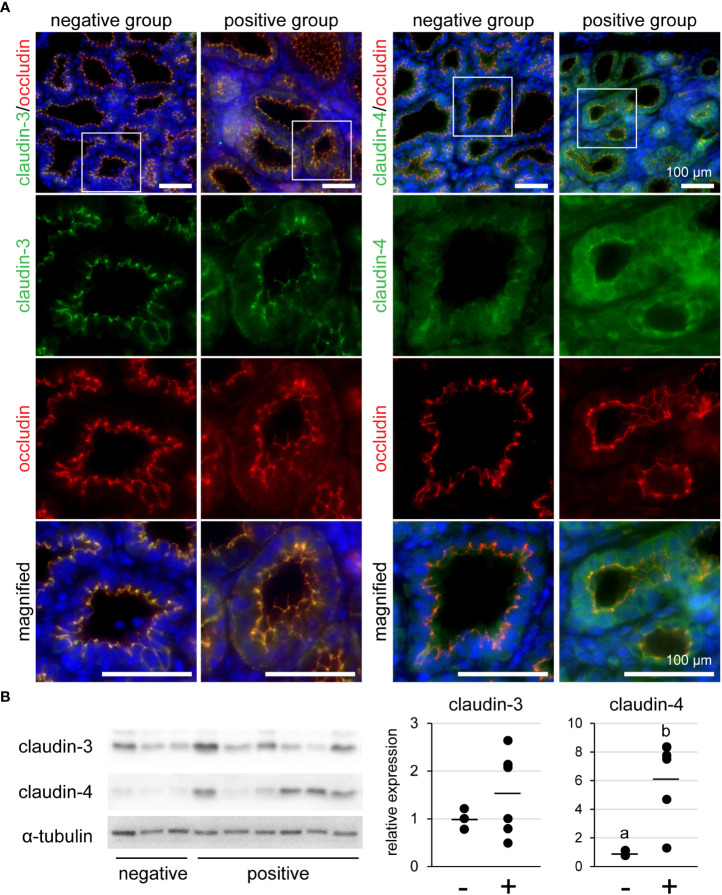
**(A)** Representative images of immunofluorescence against claudin-3 and claudin-4 in tertiary lymphoid organ-positive and -negative goat mammary gland tissues. Occludin is a marker for tight junctions. Scale bar, 100 μm. **(B)** Bands detected by western blot and the results of densitometry analyses. Alpha-tubulin serves as internal control; (−) lymphocyte aggregation-negative group; (+) lymphocyte aggregation-positive group. Different letters indicate a significant difference between groups (p < 0.05).

We investigated the localization of claudin-3 and claudin-4 in mammary glands using immunofluorescence (**Figure 5A**). Occludin was used as a marker of TJ regions in mammary epithelial cells. Claudin-3 was localized at the most-apical lateral membranes, along with occludin, regardless of the presence of lymphocyte aggregation. In contrast, claudin-4 was partly localized near occludin in the lymphocyte aggregation-negative group, but was localized along with occludin in the positive group. We used western blotting to investigate the levels of claudin-3 and claudin-4 (**Figure 5B**). Our results showed that the amount of claudin-4 in the lymphocyte aggregation-positive group (n = 6) was approximately six-fold higher than that in the negative group (n = 3) [p = 0.016].

### Influence of lymphocyte aggregation on milk components

**Figure 6:**
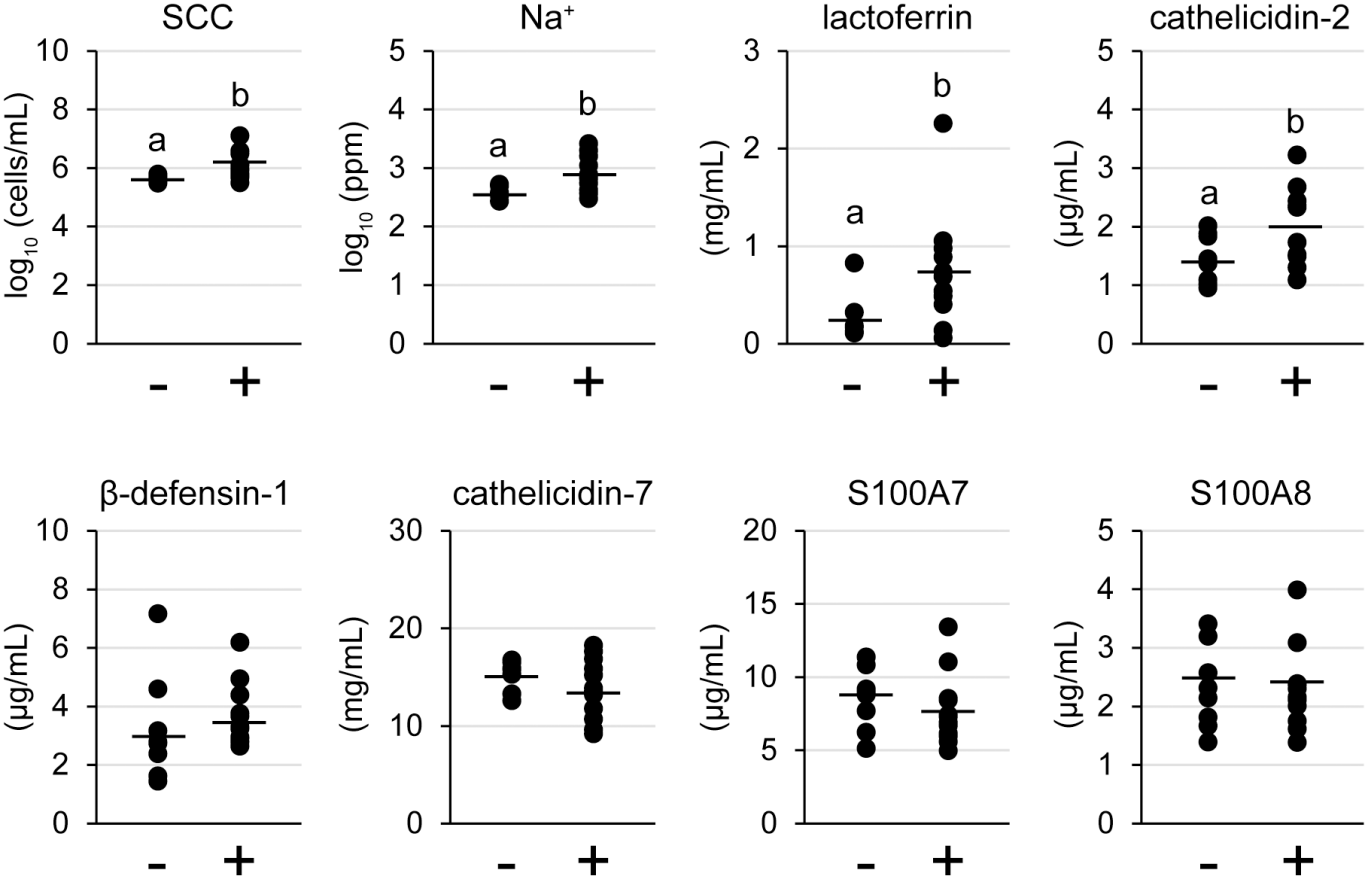
Somatic cell count (SCC), Na^+^, and antimicrobial components in tertiary lymphoid organ-positive and -negative goat milk. (−) lymphocyte aggregation-negative group; (+) lymphocyte aggregation-positive group. Different letters indicate a significant difference between groups (p < 0.05).

To reveal the relationship between the presence of lymphocyte aggregation and TJ barrier function or antimicrobial component production, we investigated the SCC, Na^+^, and antimicrobial components in milk (**Figure 6**). The levels of both SCC and Na^+^ in milk of the lymphocyte aggregation-positive group (n = 12) were significantly higher than those in the negative group (n = 8) [log10 SCC: 6.06 ± 0.46 cells/mL vs. 5.63 ± 0.13 cells/mL, p = 0.018; log10 Na^+^: 2.90 ± 0.29 ppm vs. 2.58 ± 0.13 ppm, p = 0.008, respectively]. Concentrations of both lactoferrin and cathelicidin-2 in the lymphocyte aggregation-positive group were significantly higher than those in the negative group (lactoferrin: 0.73 ± 0.57 mg/mL vs. 0.26 ± 0.40 mg/mL, p = 0.039; cathelicidin-2: 2.00 ± 0.65 μg/mL vs. 1.45 ± 0.42 μg/mL, p = 0.049). There were no statistical differences between the groups in the concentrations of S100A8, β-defensin-1, cathelicidin-7, and S100A7.

## Discussion

In this study, we investigated the presence or absence of TLOs in the mammary glands of lactating goats, focusing on the presence of T cells, B cells, HEVs, follicular dendritic cells, and the formation of germinal centers. HE staining revealed that 19 out of 30 udders had aggregations of lymphocytes regardless of udder side, age, parity, days after parturition, previous *E. coli*, or LPS infusions. The aggregates showed positive reactions against CD20 (B-cell marker), CD3 (T-cell marker), MECA79 (HEV marker), and CD40 (follicular dendritic cell marker), which were classified as stage 1 TLOs. In addition, large aggregations rarely showed separate localization of B cells and T cells, which were classified as stage 2 TLOs. The large aggregations showed a positive reaction against BCL6 (germinal center marker) but BCL6 was sparsely localized in the aggregates. In lymph nodes as secondary lymphoid organs, BCL6-positive cells were collectively localized and formed follicles (**Supplementary Figure 1**). Thus, our findings suggest that a majority of the udders of lactating goats have stage 1 TLOs, and occasionally have stage 2 TLOs.

The formation of TLOs contributes to local immunoglobulin production. In the nasal cavities, the TLO-positive group showed a larger number of IgG- or IgA-positive cells than the negative group (5). Furthermore, in cardiac allograft vasculopathy, the TLO-positive group also showed a larger number of IgG- or IgM-positive cells (30). In this study, the TLO-positive group showed significantly higher concentrations of IgG and IgA in milk and a larger number of IgA-positive cells. Thus, these findings suggest that TLOs contribute to local immunity in the mammary glands by producing IgG and IgA. In contrast, *E. coli*-specific IgG and IgA titers were not significantly different between the lymphocyte aggregate-positive and -negative groups. *E. coli* is an environmental mastitis-causing pathogen (31). The formation of TLOs observed in this study may not be due to *E. coli* infection. In TLOs of the lungs in mice, pathogen-specific IgG or IgA titers are increased by pathogen infection (4). Therefore, specific antigen presentation along with adjuvants to TLOs may induce pathogen-specific immunoglobulins. Another reason is that the TLOs observed in this study were immature. Mature TLOs form germinal centers. To develop the TLOs, repeated antigen exposure or chronic inflammation is required (32, 33). Further study is needed to reveal how to induce maturation of TLOs in mammary glands, and how to induce formation of the germinal center and produce antigen-specific immunoglobulins.

TJs regulate the permeability of small molecules via paracellular pathways. In TLO-positive mammary glands, claudin-4 was localized at the TJ region indicated by occludin, and its amount was also higher than that in the negative group. The presence of claudin-4 in the TJ region or an increase in the amount is involved in the weakening of TJ barrier function in lactating mammary glands (18, 19). In addition, the SCC and Na^+^, indicators of the TJ barrier function (34, 35), were also higher in the milk of the positive group than in that of the negative group. Normally, Na^+^ concentration in blood is higher than in milk. Lactating mammary epithelial cells form less-permeable TJs and prevent the leakage of Na^+^ into milk. However, the TJs in mammary glands with TLOs were weakened, resulting in an increase in Na^+^ concentration in milk. The SCC is also increased with TJ permeability. Thus, TLO-positive mammary glands have leakages in the TJs, which induces the formation of TLOs by increasing exposure to antigens.

The lactoferrin and cathelicidin-2 concentrations in the milk of the positive group were significantly higher. These components are produced by leukocytes, which migrate into the mammary lumen (28, 36). Thus, it suggests that lactoferrin and cathelicidin-2 increased with the increase in SCC. In contrast, the presence of TLOs did not affect concentrations of other antimicrobial components in milk. In psoriasis, which is a chronic inflammation of the skin, S100A7 is overexpressed (37). If a specific marker for TLOs in mammary glands is found, it may be possible to classify livestock and manage mastitis based on this classification. We revealed that mammary glands with TLOs show high concentrations of lactoferrin and cathelicidin-2, which may contribute to local immunity with immunoglobulins.

In this study, we investigated lactating goat mammary glands for the presence of TLOs and found that most udders had stage 1 TLOs. In addition, TLOs in mammary glands contribute to local immunity by producing immunoglobulins, lactoferrin, and cathelicidin-2. The formation of TLOs is usually associated with chronic inflammation (6). Mammary glands with TLOs show leakage of TJs, which may increase exposure to antigens. Considering milk production in milk-producing ruminants, the formation of TLOs by chronic inflammation causes a decrease in milk yield or quality but is expected to increase local immunity. In addition, it has been reported that lymphocyte aggregation exists in the mammary glands of sheep even at puberty (2). Further research is needed to reveal the mechanism of formation of TLOs in mammary glands focused on repeated antigen stimulation, chronic inflammation, or time of emergence and to establish a method that simultaneously studies milk production and introduction of TLOs.

## Data availability statement

The original contributions presented in the study are included in the article/**Supplementary Material**. Further inquiries can be directed to the corresponding author.

## Ethics statement

The animal study was reviewed and approved by the Animal Research Committee of Hiroshima University (No. C14-5). Written informed consent was obtained from the owners for the participation of their animals in this study.

## Author contributions

NI, YT, and SN conceived the study and designed the experiments. YT and SN collected and analyzed data. YT wrote the first draft of the manuscript. NI provided project administration and supervision. All authors contributed to the article and approved the submitted version.

## Acknowledgments

We thank Yukinori Yoshimura, Masayuki Shimada, and Takashi Umehara at the Graduate School of Integrated Sciences for Life, Hiroshima University, for their helpful advice and technical assistance with our experiments.

## Conflict of interest

The authors declare that the research was conducted in the absence of any commercial or financial relationships that could be construed as a potential conflict of interest.

## Publisher’s note

All claims expressed in this article are solely those of the authors and do not necessarily represent those of their affiliated organizations, or those of the publisher, the editors and the reviewers. Any product that may be evaluated in this article, or claim that may be made by its manufacturer, is not guaranteed or endorsed by the publisher.

## Supplementary material

**Supplementary Figure 1** Representative images of hematoxylin and eosin (HE) staining and immunohistochemistry against CD20 (B cells), CD3 (T cells), MECA79 (high endothelial venules; HEVs), CD40 (follicular dendritic cells), and BCL6 (germinal center) in lymph nodes of lactating goats. Scale bar, 100 μm.

**Supplementary Figure 2 (A)** Representative images of hematoxylin and eosin (HE) staining and immunohistochemistry against MECA79 (high endothelial venules; HEVs). Images of the same position are shown. Representative images of immunofluorescence against MECA79 and claudin-5 in lymph nodes (B) or mammary glands **(C)** of lactating goats. Claudin-5 is a marker for blood vessels. Scale bar, 100 μm.

**Supplementary Figure 3 (A)** Representative images of negative control for immunofluorescence against secondary antibodies. Scale bar, 100 μm. **(B)** Original membranes of western blotting. Black frames are clipped for **Figure 5B**.
