# Supplementary figures and images for: Investigating mammary glands of lactating goats for the presence of tertiary lymphoid organs
Source: Front Immunol. 2022 Aug 10;13:941333. doi: 10.3389/fimmu.2022.941333 (PMC9399771; doi:10.3389/fimmu.2022.941333)

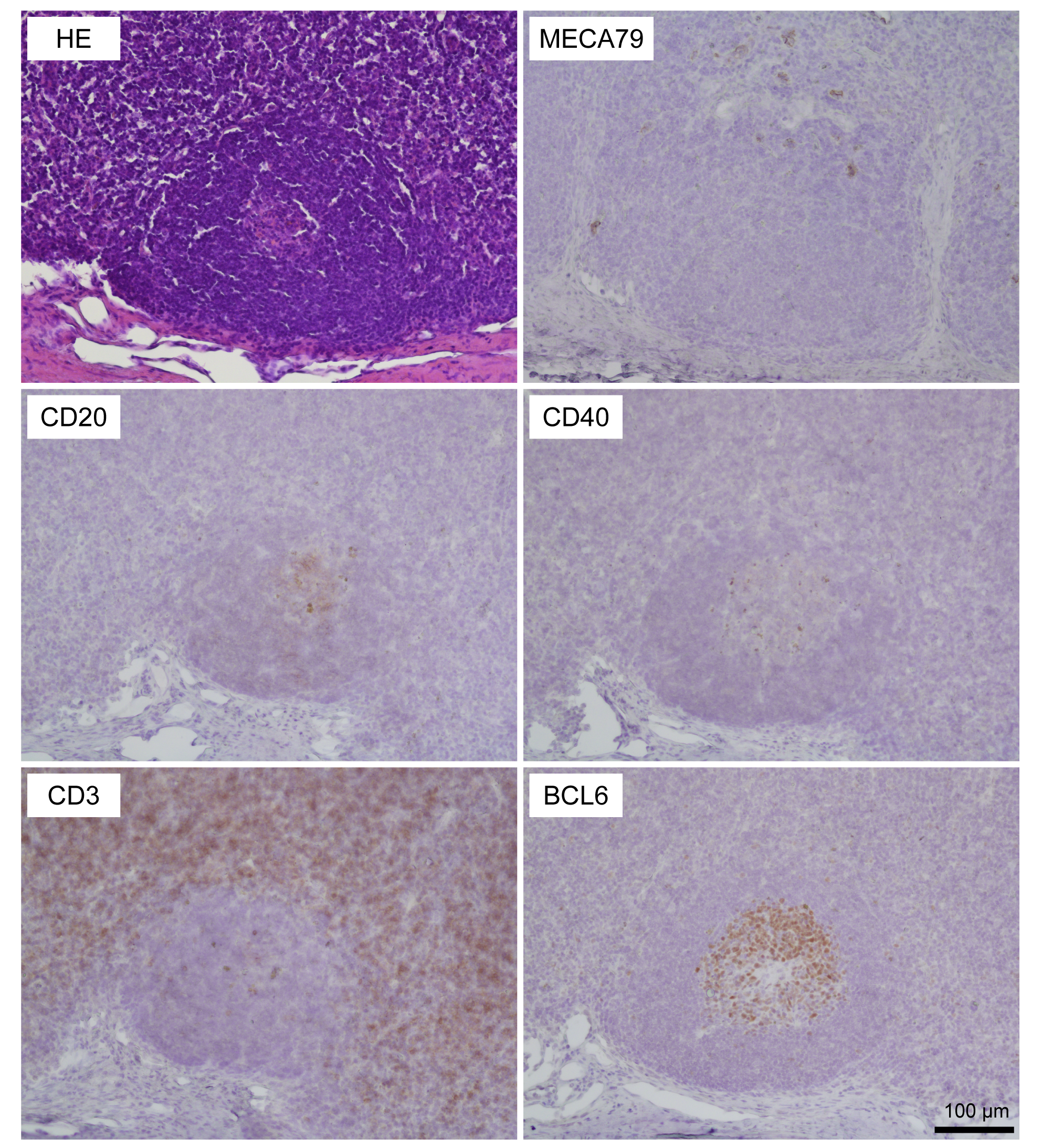

Supplement: Supplementary file 1 — Supplementary Figure 1 Representative images of hematoxylin and eosin (HE) staining and immunohistochemistry against CD20 (B cells), CD3 (T cells), MECA79 (high endothelial venules; HEVs), CD40 (follicular dendritic cells), and BCL6 (germinal center) in lymph nodes of lactating goats. Scale bar, 100 μm. [file Image_1.tif]

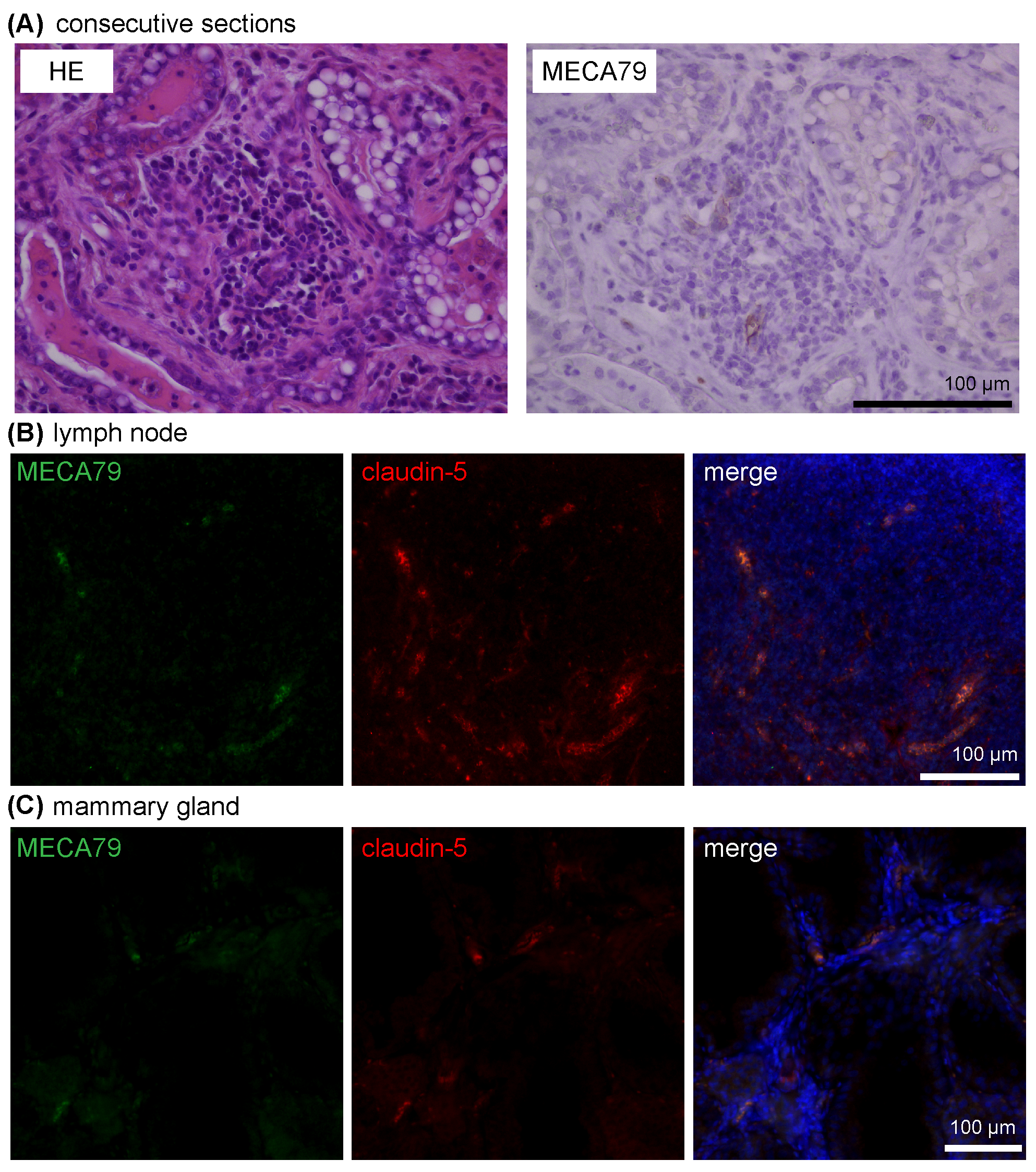

Supplement: Supplementary file 2 — Supplementary Figure 2 (A) Representative images of hematoxylin and eosin (HE) staining and immunohistochemistry against MECA79 (high endothelial venules; HEVs). Images of the same position are shown. Representative images of immunofluorescence against MECA79 and claudin-5 in lymph nodes (B) or mammary glands (C) of lactating goats. Claudin-5 is a marker for blood vessels. Scale bar, 100 μm. [file Image_2.tif]

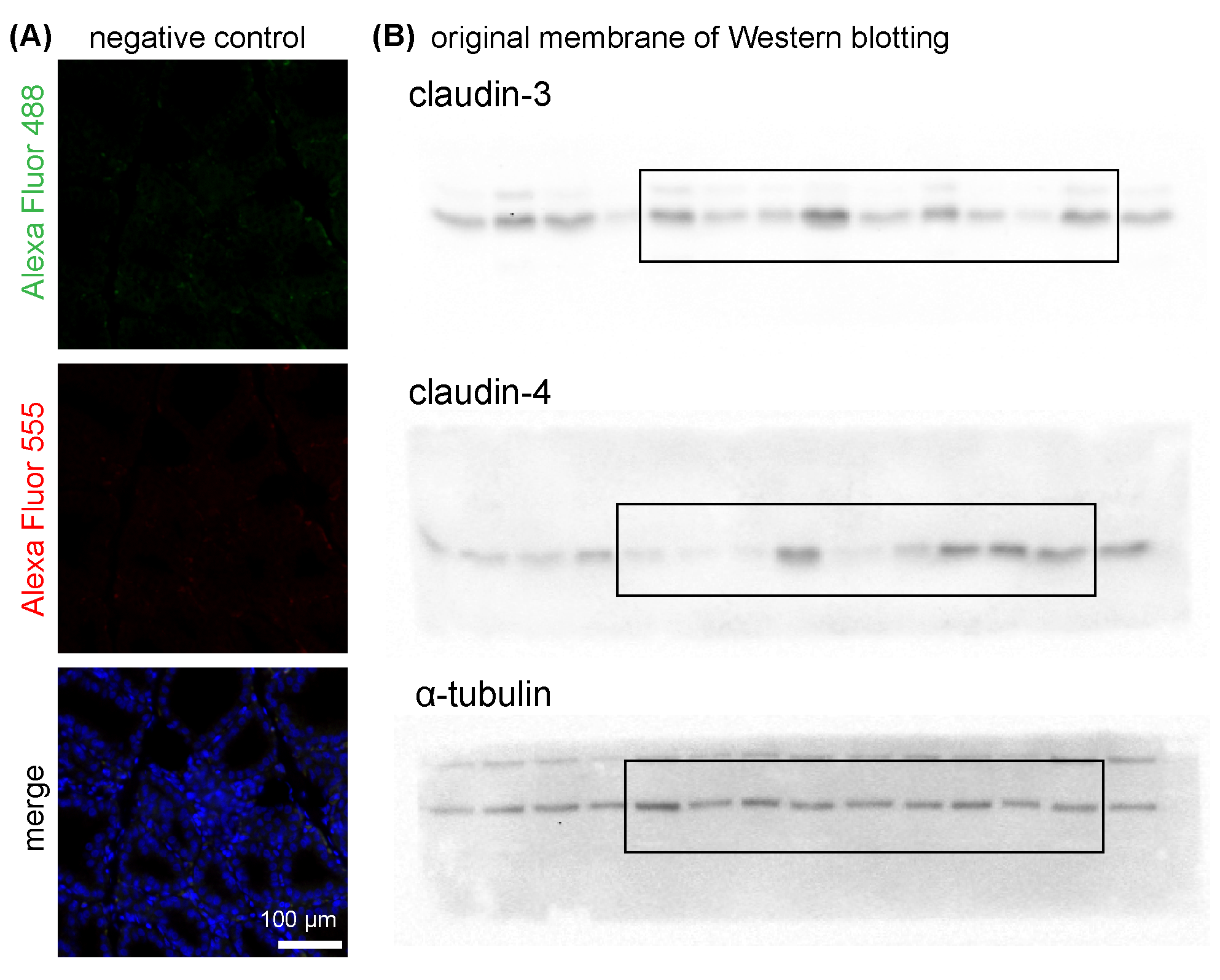

Supplement: Supplementary file 3 — Supplementary Figure 3 (A) Representative images of negative control for immunofluorescence against secondary antibodies. Scale bar, 100 μm. (B) Original membranes of western blotting. Black frames are clipped for Figure 5B . [file Image_3.tif]
